# Disseminated Sporotrichosis With Brain Abscesses in an HIV-Infected Patient

**DOI:** 10.7759/cureus.8016

**Published:** 2020-05-07

**Authors:** Nathaniel Parker, Nora Strong, Pie Pichetsurnthorn, Daniel Lalich, Thomas Moore

**Affiliations:** 1 Internal Medicine, University of Kansas School of Medicine, Wichita, USA; 2 Pathology, Wesley Medical Center, Wichita, USA; 3 Infectious Disease, University of Kansas School of Medicine, Wichita, USA

**Keywords:** sporothrix schenckii, sporothrix schenckii species complex, disseminated sporotrichosis, brain abscess, hiv aids

## Abstract

Sporotrichosis is a mycotic infection caused by the Sporothrix schenckii species complex. It typically presents as a localized, mostly cutaneous or lymphocutaneous infection, but has been increasingly presenting as a disseminated infection in immunocompromised individuals, especially in HIV/AIDS patients. We also included a literature review of sporotrichosis in this patient population. The aim of this case report is to raise awareness about atypical presentations of sporotrichosis in an attempt to decrease the time to diagnosis, initiate treatment earlier when infection is suspected, and improve overall survival in vulnerable patient populations.

## Introduction

Historically, sporotrichosis referred to a disease caused by the single species, *Sporothrix schenckii* [1]. However more recently, morphologically similar, but taxonomically diverse sibling species have emerged. *S. schenckii,* now recognized as the *S. schenckii *species complex, consists of five distinct species: *S. schenckii sensu strictu*, *S. brasiliensis*, *S. globosa*, *S. mexicana* and *S. luriei* [2]. Aggregated for their pathogenicity and virulence, these phylogenetic species are capable of causing acute or chronic subcutaneous and disseminated mycosis [1].

Sporotrichosis is highly prevalent. It occurs globally with higher prevalence observed in tropical and subtropical regions, namely Japan, China, Australia, Mexico, Brazil, Colombia, Peru and India [1]. Historically, transcutaneous traumatic implantation was thought to be the predominant mode of transmission, but rare cases of pulmonary and disseminated sporotrichosis due to fungal spore inhalation have been reported [3]. Despite no predilection for age, gender, or race, certain populations are more susceptible to *Sporothrix *species infection. Immunocompromised individuals are at an increased risk of developing disseminated sporotrichosis [4]. Moreover, clinically significant lymphocutaneous and disseminated forms of sporotrichosis have recently become more prevalent in HIV-infected patients, suggesting an emerging health concern among the global HIV/AIDS population [5].

This report presents a rare case of osteoarticular sporotrichosis with dissemination to the brain in an HIV-infected patient from a non-endemic region. Given the risk of opportunistic infections in this population, clinicians should have a low threshold of suspicion for atypical systemic mycoses such as disseminated sporotrichosis.

## Case presentation

A 30-year-old male presented to the hospital for new-onset seizures, left hand erythema with tenderness, and right knee swelling. He was diagnosed with acquired immunodeficiency syndrome (AIDS) three years prior with inconsistent use of his home prophylactic trimethoprim / sulfamethoxazole medication and antiretroviral therapy. Two years prior to presentation, he was diagnosed with septic arthritis of the third digit on the left hand. Synovial fluid cultures grew *S. schenckii* and he was discharged on itraconazole; however, he had not been compliant. He was readmitted to the same hospital for septic arthritis of the left knee, cultures grew *S. schenckii *again, and the patient was instructed to restart itraconazole. However, he continued to struggle with medication compliance. Of note, he has not been to any endemic area for fungal infections.

**Figure 1:**
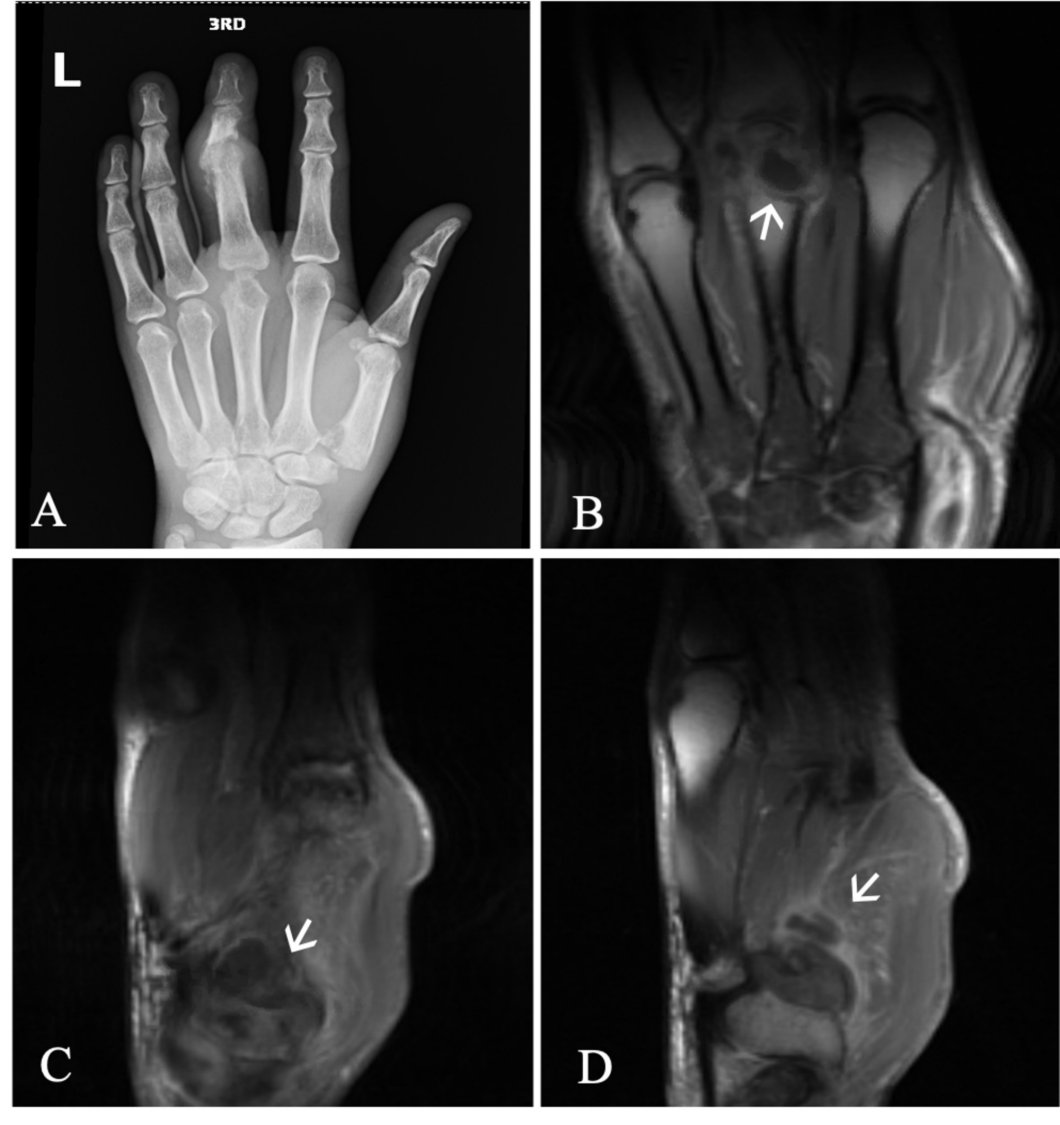
Left hand imaging demonstrates multiple areas concerning for infectious and inflammatory arthritis, tenosynovitis, tophus, intraosseous abscess, and osteomyelitis. (A) Left hand X-ray exhibits multifocal areas of bone erosion, primarily affecting the third metacarpophalangeal (MCP) and proximal interphalangeal joints, base of the thumb, and distal radius. (B) Contrast-enhanced multiplanar, multisequence MRI of the left hand demonstrate the third MCP joint is distended with fluid, T1 hypointense signal and marrow edema in the adjacent metacarpal and proximal phalanx, and a peripherally enhancing fluid collection in the third metacarpal head or intraosseous tophus (arrow) measuring 9 x 12 mm. (C) The first metacarpal and trapezium show diffuse marrow edema and T1 hypointense signal, joint recesses are distended with fluid, erosion of the head of the first metacarpal exists along its ulnar aspect, and a peripherally enhancing focus (arrow) exists in the base of the first metacarpal measuring 10 x 12 mm. (D) Fluid distention of the third MCP joint, third extensor digitorum tendon sheath, and first carpometacarpal joint (arrow) likely represent infectious or inflammatory arthritis and tenosynovitis.

**Figure 2:**
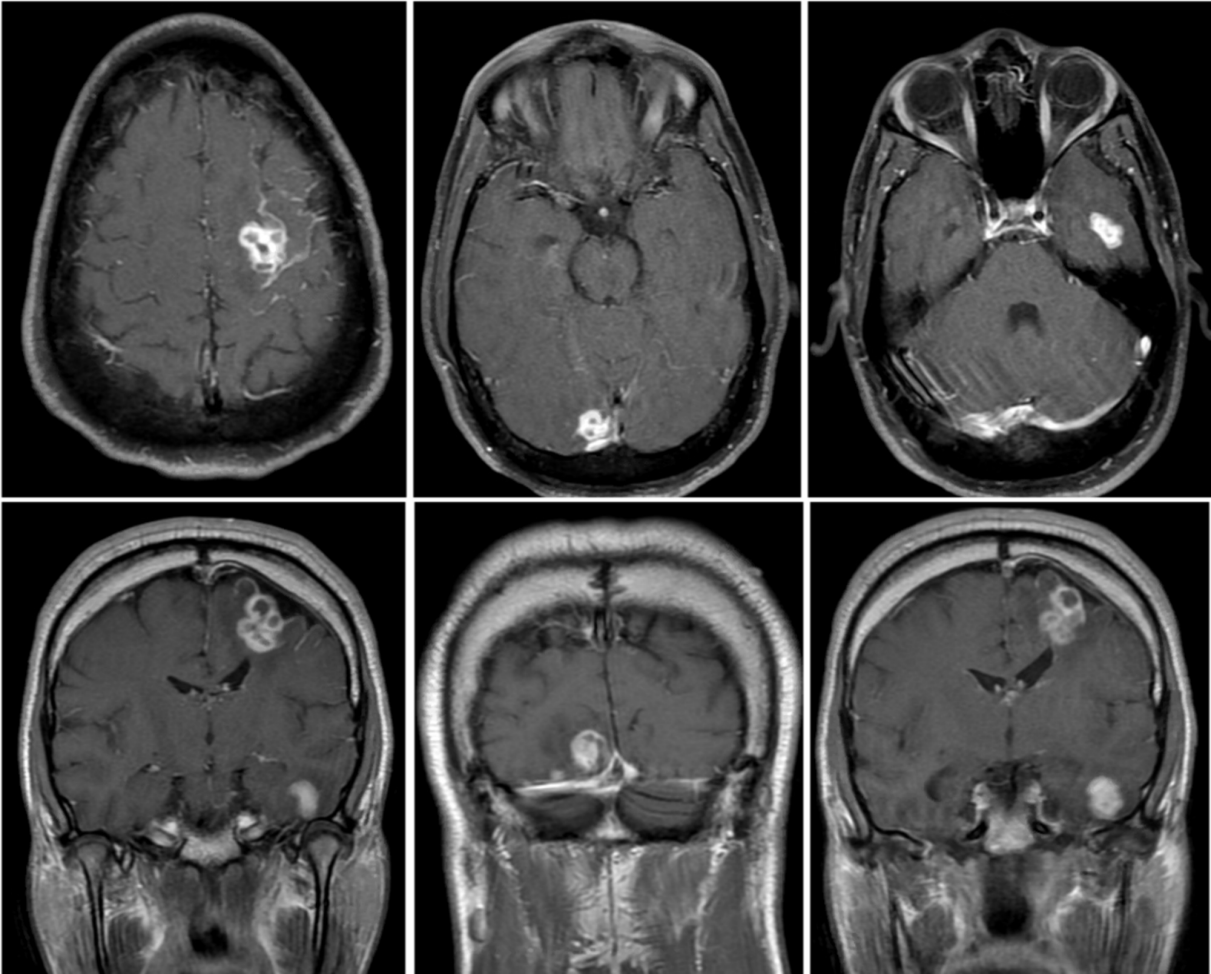
Brain MRI demonstrates multifocal enhancing lesions concerning for metastasis versus infection or abscesses. Enhancing lesions in the left superior frontal lobe (2.0 x 1.9 x 2.4 cm), left temporal lobe (1.7 x 1.3 x 1.7 cm), and right inferior occipital lobe (1.3 x 1.4 x 1.7 cm) with adjacent T2 hyperintense signal abnormality and mild perilesional mass effect. Lesions demonstrate faint peripheral diffusion restriction, without central diffusion restriction.

**Figure 3:**
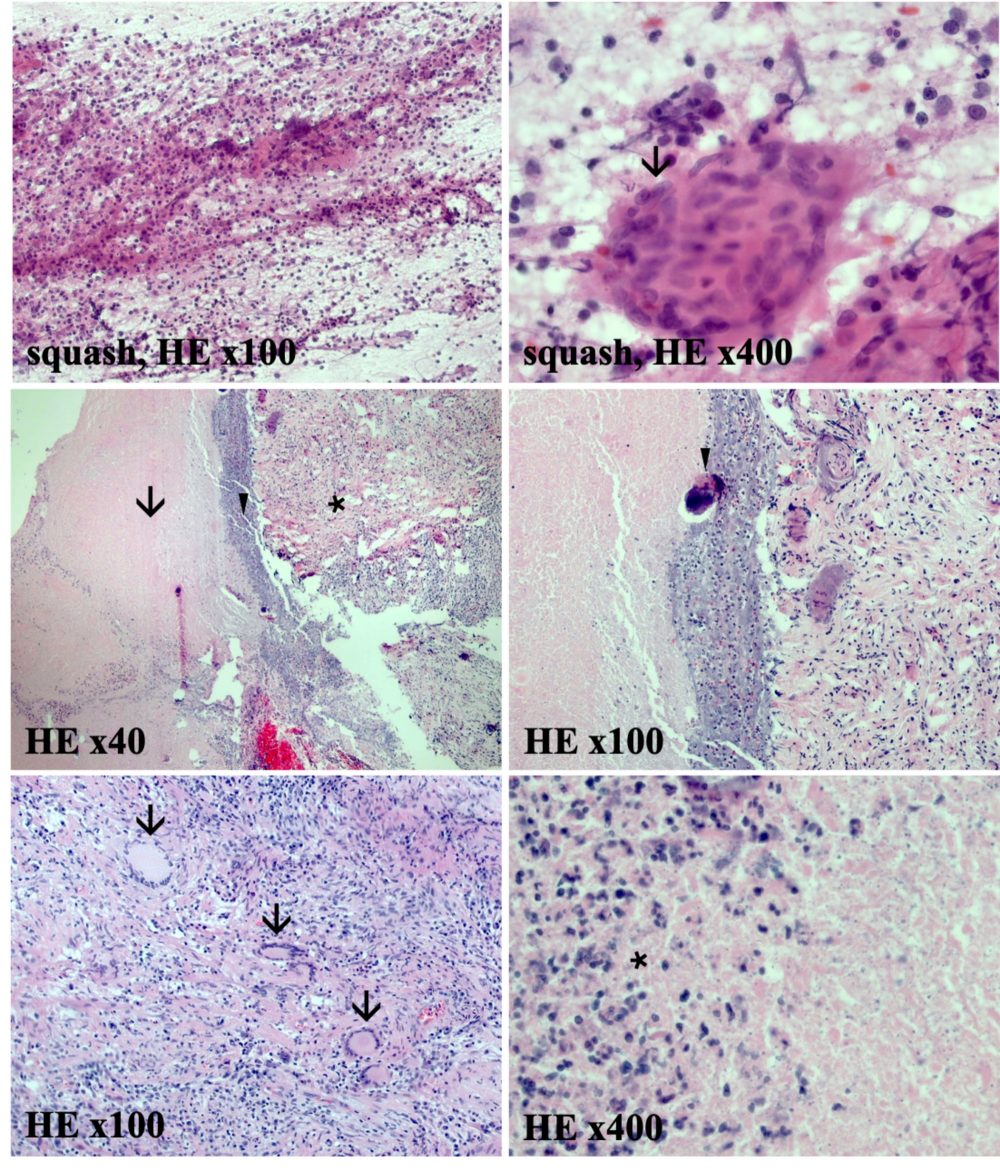
Intraoperative cytology by squash preparation of the resected left parietal brain mass is negative for malignant central nervous system tumors. Extensive neutrophilic infiltration with granulomatous giant cells (arrow) is evident (squash, HE stain x100 and x400). Surgical pathology specimens show acute abscesses and marked necrosis. Fibrotic tissue (arrow), dense fibrinoid necrosis and inflammatory debris (arrowhead), and a substantial neutrophilic infiltration (asterisk) within the brain parenchyma (HE stain, x40). Abscesses containing Langerhans giant cells (arrows), fibrosis, dystrophic calcifications (arrowhead), and a remarkable neutrophilic infiltration (asterisk) at higher magnifications (HE stain, x100 and x400).

**Figure 4:**
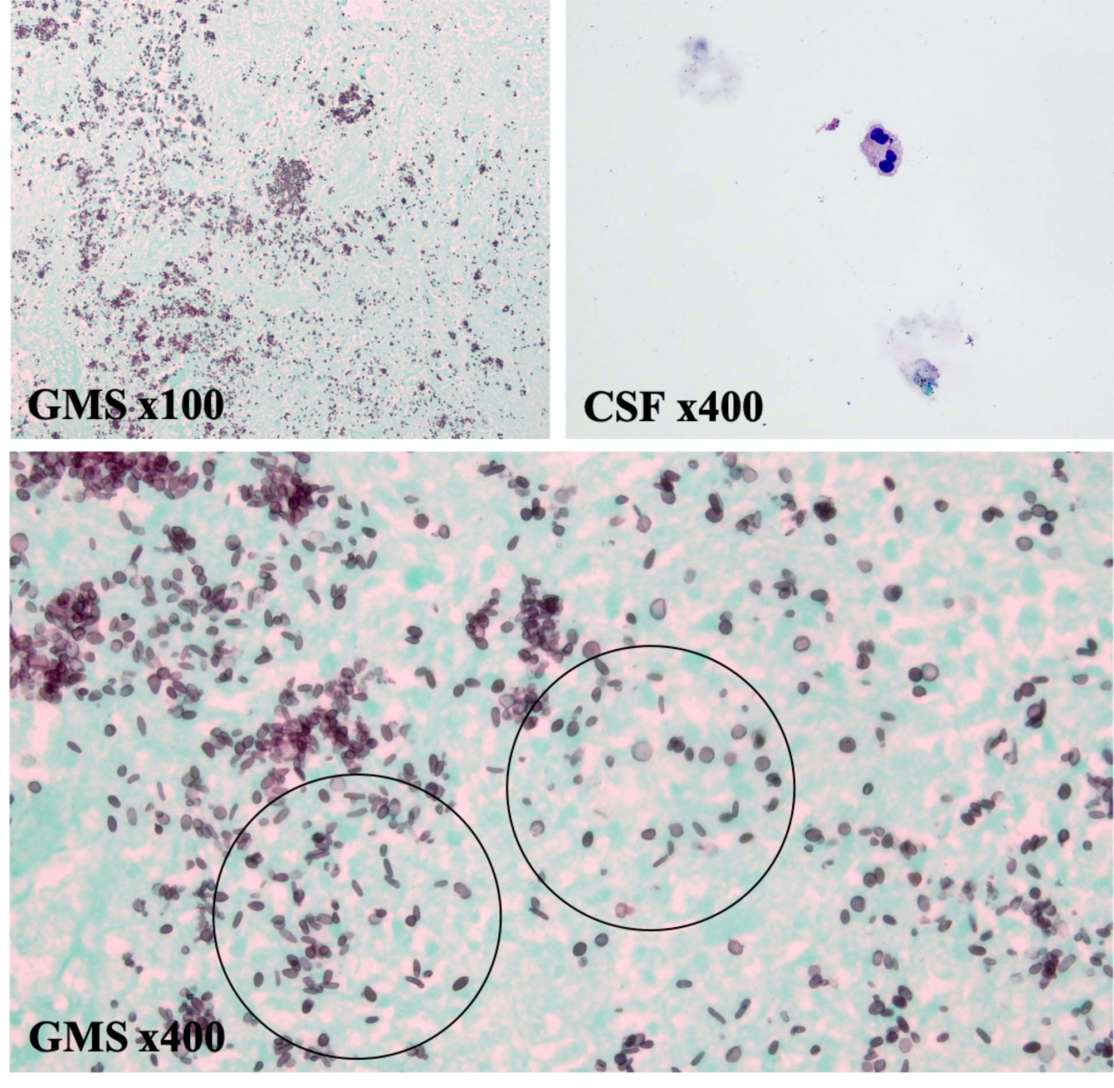
The surgical pathology of the resected left parietal brain mass tissue suggests disseminated S. schenckii infiltrating brain tissue. Grocott's methenamine silver (GMS) staining positive for abundant oval, spherical, and cigar-shaped fungal yeast (circled). Yeast identified most heavily in the necrotic areas. Clinical history of chronic monoarticular arthritis due to sporotrichosis, GMS staining, and HE histologic findings consistent with *S. schenckii *(GMS x100 and x400). Cerebrospinal fluid (CSF) cytology shows scattered red blood cells, with rare neutrophils, monocytes (not pictured), and lymphocytes (not pictured). No cytologically malignant cells (Wright-Giemsa stain labeled as CSF x400) identified. Flow cytometry performed on resected brain mass tissue showed a nondiagnostic polyclonal B-cell and NK-cell populations (dot plot not pictured).

On admission, laboratory testing was notable for CD4+ count of 64 cells/µL and HIV-1 viral load of 247 copies/mL. Left hand imaging was suspicious for acute on chronic osteomyelitis primarily originating from the 3^rd ^digit of the left hand (Figure 1). Magnetic resonance imaging (MRI) of the brain with contrast was obtained, and showed multifocal irregular ring-enhancing lesions with surrounding edema (Figure 2). Given his history of HIV infection, the differential diagnosis now included intracranial toxoplasmosis, cryptococcosis, histoplasmosis, aspergillosis, tuberculosis, and primary central nervous system (CNS) lymphoma. However, when considered with the patient’s clinical presentation and history, sporotrichosis disseminated to the CNS was more likely. Cerebrospinal fluid (CSF) analysis by lumbar puncture was non-diagnostic. Craniotomy was performed for mass resection and diagnosis. Intraoperative cytology by squash preparation of the resected left parietal brain mass was negative for a primary or secondary CNS malignancy (Figure 3, squash HE). Immunohistochemical (IHC) staining of the resected brain abscess revealed an extensive neutrophilic infiltration and necrosis (Figure 3, HE). Grocott's methenamine silver (GMS) staining was positive for abundant oval, spherical, and cigar shaped yeast in the sampled tissue (Figure 4, GMS).

Gram stain of left parietal brain abscess specimens only showed a few neutrophils and fungal elements. No acid-fast bacilli (AFB) were seen on AFB smear, fluorescent staining, and culture. No fungal elements were observed on fungus smear or culture. Gram stain of right parietal brain abscess specimens showed few neutrophils only. No organisms or fungal elements were observed on gram stain or smear. In contrast to the aforementioned specimens, two out of two fungal cultures were positive for S. schenckii colonies. Histopathology and gram stain on CSF fluid was primarily acellular with no bacteria or fungi isolated (Figure 4, CSF). CSF meningitis/encephalitis panel was negative. Serum immunologic testing for cytomegalovirus, as well as bacterial and fungal cultures, was negative. This IHC, special staining, and microbiologic profile supported disseminated *S. schenckii *to the brain, rather than a primary or secondary CNS malignancy, or another opportunistic infection.

His home antiretroviral medication (bictegravir / emtricitabine / tenofovir) was resumed, and trimethoprim / sulfamethoxazole was started prophylactically. Oral itraconazole was continued until *S. schenckii *was identified in the brain via culture. Intravenous liposomal amphotericin B dosed at 5 mg/kg/d (or 350 mg per dose) was started and continued for six weeks. He was transitioned to twice-daily oral itraconazole 200 mg on the day of hospital dismissal with instructions to continue for one year, then de-escalate to a once daily regimen indefinitely.

## Discussion

Sporotrichosis, classically known as rose gardener’s disease, is the mycotic infection of humans and other mammals by a dimorphic fungus termed the *S. shenckii *species complex [2]. Cutaneous, lymphocutaneous, and articular presentations are well documented, with disseminated cases occurring primarily in immunocompromised individuals. Sporotrichosis has been reported as isolated cases or in reference to outbreaks, sometimes related to specific occupational exposure [6,7]. The incidence of disseminated sporotrichosis is difficult to determine but increased clinical awareness of sporotrichosis allows for earlier detection and treatment, potentially prolonging survival. One reason for uncertainty regarding the incidence could stem from the organism’s predilection to joint inoculation, particularly in the hand. Arthritis caused by *S. schenckii*, similar to most inflammatory arthropathies of the hand and wrist, is often difficult to investigate by fluid collection for culture [8]. Incubation on agars typically induces colony formation after 14 days [2,4,8,9]. But a 30-day observation may be necessary before declaring cultures negative [10]. The gold standard for sporotrichosis diagnosis remains culture [8].

Unfortunately, the time required for culture is not conducive to prompt treatment of infection and non-suppurative lesions provide little material for culture. For this reason, non-culture techniques have been developed to improve the rate of *S. schenckii *diagnosis. These methods could provide a vital diagnostic tool in the setting of negative cultures, low fungal burden, or specimen crowding due to co-infection [8]. Polymerase chain reaction amplification assays have demonstrated high specificity. Early identification of avirulent and virulent strains is paramount for risk stratification and management during outbreaks of endemic illnesses. The sensitivity and specificity of intradermal sporotrichin skin testing have been shown to be 94.5% and 95.2%, respectively [11]. However, this method is not reliable since it is unable to differentiate between acute and prior infections [11,12]. Detection of IgG antibodies against various *Sporothrix* antigens has resulted in 97% sensitivity and 89% specificity [13]. Unfortunately, the limitations are similar to other antibody-based assays [14].

*Sporothrix* is often difficult to identify by histological examination. Asteroid bodies, which are extracellular budding yeast surrounded by eosinophilic spicules, have a sensitivity of about 94% [15]. Direct visualization of asteroid bodies, the Hoeppli-Splendore phenomenon, is unlikely as less than 50% of histopathology specimens demonstrate this and they are non-specific [15]. However, clinical correlation paired with demonstration by standard histopathological, immunohistochemical techniques, or special stains like the GMS stain significantly increases sensitivity and specificity for the diagnosis of sporotrichosis [8].

The increasing number of reports of sporotrichosis in the HIV-infected population has been postulated to represent an emerging global health concern associated with the AIDS pandemic [5]. To date, less than 300 unique cases of disseminated sporotrichosis affecting the CNS in the HIV-infected population have been published [5,6,12,16-18]. According to estimates of prevalence from case studies, the true burden of disseminated sporotrichosis to the CNS in HIV-infected patients may not be adequately illustrated. After a thorough review, no prior cases of brain abscesses secondary to sporotrichosis have been reported in the literature. To the best of our knowledge, this is the first case of CSF-negative, leptomeningeal-negative, and culture-confirmed *S. schenckii* that presented as multifocal abscesses in an HIV-positive patient.

Although sporotrichosis causes considerable morbidity, it rarely disseminates to the CNS and causes mortality [3]. In two of the largest reviews of sporotrichosis in HIV‑infected patients, Moreira et al. and Freitas et al. revealed disseminated sporotrichosis to be a significant concern in HIV/AIDS patients. Zoonotic transmission by infected cats occurred primarily in hyperendemic areas such as Brazil. CD4+ cell counts below 200 may increase the likelihood of disseminated sporotrichosis, with CNS involvement rarely being the presenting symptom. However, CD4+ cell counts below 100 could potentiate the risk of *S. schenckii *disseminating to the CNS. In the HIV-infected population, CNS involvement of *S. schenckii *was more common and associated with poorer outcomes [6]. Of those that suffered from disseminated forms of the disease, *S. brasiliensis* was the pathogenic agent in a substantial portion of HIV-infected sporotrichosis patients. Moreira et al. and Freitas et al. showed that HIV-positive patients are more susceptible to disseminated forms of sporotrichosis and recommended screening and/or serial monitoring lumbar punctures for every patient with sporotrichosis and HIV. Finally, *S. schenckii *should be considered to be a serious opportunistic pathogen in all patients with HIV/AIDS [6,9,16]. Resuming antiretroviral therapy in HIV-positive patients diagnosed with disseminated sporotrichosis remains controversial. Moreira et al. recommended delaying antiretroviral therapy if CNS disease is present, CD4+ cell count is low, and viral load is elevated [6].

This report supports the potential for aggressive infection by *S. schenckii *in immunosuppressed patients, thus making early identification and initiation of antifungal treatment paramount. Unfortunately, data regarding antifungal susceptibilities is sparse, as testing is rarely performed. Different *Sporothrix *strains have been reported to have different antifungal susceptibilities, especially in HIV-positive patients [19]. Initial amphotericin B and itraconazole therapy, followed by long-term itraconazole monotherapy is the most common and effective antifungal regimen implemented worldwide for sporotrichosis [6].

## Conclusions

Here, we describe the first case report to our knowledge of CSF-negative, leptomeningeal-negative, and culture-confirmed* S. schenckii *that disseminated to the brain and presented as discrete multifocal abscesses in an HIV-positive patient. When HIV-infected patients complain of concomitant cutaneous, osteoarticular, and neurologic symptoms, disseminated sporotrichosis should be considered. HIV-infected patients are at an increased risk for developing not only disseminated forms of the disease, but also CNS involvement, which significantly worsens prognosis. As a result, increased clinical awareness of sporotrichosis allows for earlier detection and treatment, potentially prolonging survival.
